# The synergistic role of viral infection and immune response in the pathogenesis of facial palsy

**DOI:** 10.1007/s13365-025-01258-7

**Published:** 2025-05-15

**Authors:** Aijun Wang, Wei Xie, Jian Zhang

**Affiliations:** Quzhou hospital of TCM, Quzhou, China

**Keywords:** Viral infection and facial palsy, Immune-viral synergy, Neuroinflammation, Viral latency and reactivation

## Abstract

Facial palsy refers to facial muscle paralysis and is typically brought about by viral infections, such as herpes simplex virus type 1 (HSV-1), herpes zoster virus (VZV), and SARS-CoV-2. While significant progress has been achieved in viral facial palsy pathogenesis, mechanisms of viral infection-immunity synergy are yet to be revealed. The authors of this article made an attempt to fill this gap by critically summarizing how viral infection causes inflammation and damage to the facial nerve through an immune response mechanism in the facial palsy pathogenesis. We also summarize the current treatment modalities and their respective efficacies. The article set the conditions under which viral infections caused by HSV-1, VZV, SARS-CoV-2, HIV, and EBV lead to facial paralysis and how the viruses infect the facial nerve, initiate an immune response, and cause nerve death. The impact involved direct viral invasion of neurons, immune evasion and induction of neuroinflammation. The review also discusses the primary role of T cells, B cells and innate immune cells in inducing or relieving the condition. The study emphasizes the need to understand the synergic effect of viral infection and immuneresponse of facial palsy as the foundation of the creation of more potent therapeutic strategies. The paper provides a detailed overview of complex interaction of immuneresponse and viral infection of facial palsy with significant level of importance regarding future research and clinical application.

**Supplementary Information:**

The online version contains supplementary material available at 10.1007/s13365-025-01258-7.

## Introduction

Facial palsy, or “facial nerve palsy,” is mainly defined as a form of paralysis that is linked with facial expression muscles. Paralysis occurs on one side of the face and can result in an inability to close or open eyes fully. Furthermore, superficial furrows are present in the nose region, and mouth corners hang loose. The most frequent cause of this condition is Bell’s palsy, which is responsible for around 60–75% of such cases(Kirchgassner et al. 2024). It is estimated that in the United States alone around 40,000 people have Bell’s palsy every year, and one person in every 60 is most likely to suffer from it during their lifetime(Hwang et al. 2025; Ogawa et al. 2025). The condition can affect any age group of people but appears to affect people between the ages of 15 to 60 years mostly. Both males and females appear to be affected by it equally. Apart from leading to a failure of facial function and aesthetics, the condition can lead to serious psychological as well as social problems in the patient. There are times when the condition recurs or results in severe complications, possibly requiring surgery or cosmetic interventions. These complications can have a tremendous impact on one’s quality of life(Alamodi et al. 2024; Bayram et al. 2024; Edalati et al. 2025).

In the last few years, the science of molecular biology and immunology has produced so much evidence that viral infection is the cause of facial palsy. Herpes of the mouth is due to herpes simplex virus (HSV-1) and is implicated in the inflammation of the facial nerve(Murphy et al. 2024; Alberts et al. 2025). Studies have proved that HSV-1 may cause inflammation and injury of the facial nerve and thus facial palsy(Giacobbe et al. 2022). There is a virus as well, herpes zoster virus (VZV), which causes chickenpox and shingles and even facial palsy. Once the body has been infected with VZV, the virus will remain latent in the body’s ganglion and will come back again someday, especially if one has a weak immune system. That is what would reactivate and cause inflammation and hence facial paralysis. VZV is the most frequent etiologic virus of facial palsy, and VZV inflammation is a leading cause of the disease(Navarro-Bielsa et al. 2023). SARS-CoV-2 was also found to induce facial palsy in a few pandemic COVID-19 instances with undefined mechanisms. Systemic viral inflammation is hypothesized to be associated to some degree with facial nerve dysfunction(Asaba et al. 2025). It is therefore of utmost importance to explore the correlation of immune response and virus with the onset of facial palsy. There is a need to continue researching the mechanisms through which viral infection provokes immune responses and immune responses influence the function of the facial nerve. This will be the basis for the development of better regimens in treatment. Also, research in the field of virus and immunity interaction can be a precursor to future prophylaxis of facial palsy. In the present review, attempts were made to break the interaction between viral infection and immunity and its causation of facial palsy. It will examine how these viruses lead to inflammation and nerve damage, how immune activation occurs, and what are the current treatments, their efficacy, and where they can be optimized. Maybe this will provide a clearer map for future research and clinical direction in facial palsy management.

## Mechanisms of viral infection in the pathogenesis of facial palsy

HSV-1 is neurophilic and enters the ganglion via sensory nerve endings and remains latent within them. HSV-1 can retrograde spread via nerve fibers during infection and cause neuroinflammation and damage. Latency and reactivation in facial ganglia is a significant mechanism of induction of facial palsy due to HSV-1(Moniuszko-Malinowska et al. 2021). It is capable of inducing a neuroinflammatory reaction following facial nerve infection which could result in nerve swelling and compression. This may lead to edema and compression of the nerve. Studies have shown HSV-1 infection to be accompanied by a vigorous inflammatory cell invasion and cytokine release in the facial nerve and that these inflammatory responses are a major mechanism of induction of facial paralysis(Zalagh et al. 2014; Li et al. 2015). The main route of transmission of VZV is respiratory droplet spread, but also by direct contact with vesicle fluid or fomites of infected individuals. When the host becomes infected with VZV for the first time, the virus results in chickenpox in the host and establishes the virus in the ganglia of the nerves and stays in latent form in a nonreplicating form within the neurons(Guan et al. 2024). Latent process of VZV is complex and includes epigenetic modifications which repress the viral gene expression, which prevents the viral replication to maintain the latent state(Ben Mohamed et al. 2023). As the body’s immunity declines during this period, latent VZV reactivates. The reactivated virus multiplies in large numbers, producing inflammation or necrosis of the invaded ganglion and resulting in neuralgia(Freire de Castro et al. 2022). Virus particles travel retrogradely along the nerve fibers and induce hemorrhagic inflammation of peripheral nerves, posterior roots, and posterior root ganglia and result in redness, pain, and inflammatory reaction of the surrounding tissue.VZV infection results in inflammatory reaction of the facial nerves, swelling, and compression of the nerves, followed by facial paralysis(Gupta et al. 2021). There has been an explosive rise in facial palsy cases during the COVID-19 pandemic, and it has been contended that infection with SARS-CoV-2 may result in facial palsy through multiple mechanisms. The viral-induced inflammatory reaction in the system results in release of a gigantic number of inflammatory mediators, and these may impair the facial nerve blood supply and function(Namavarian et al. 2023). SARS-CoV-2 is neuropathophilic, and it enters the facial nerve through various routes and results in neuroinflammation and facial nerve injury. Abnormal coagulation in COVID-19 patients results in hypercoagulability of blood and formation of microthrombi, and these occlude the blood vessels supplying the facial nerve and result in facial nerve injury and facial paralysis(Mutlu et al. 2021). HIV infection results in immunedysfunction, and the patient becomes susceptible to opportunistic infections and neuropathy. General clinical presentation of HIV-infected patients with facial palsy has closely been linked with virus-induced neuroinflammation and immunosuppression. HIV-induced facialis palsy is caused because of the infection of the nervous system by HIV, thereby leading to nerve damage and neuroinflammation(Kaplan et al. 2009). EBV is a double-stranded DNA virus that exists mainly in salivabearing fluids and causes an array of diseases. EBV also activates the immune system during infection and releases high titers of inflammatory mediators and cytokines, which can cause facial paralysis by facial nerve inflammation and edema through bystander effect or autoimmune response(Kaplan et al. 2009)(Table 1).

The main three direct viral modes of neural infection include bloodstream transmission, retrograde neuron transmission, and direct entry. Viruses infect the CNS and traverse the blood-brain barrier (BBB) via multiple mechanisms(Luo et al. 2024a). HSV infects CNS neurons through retrograde transmission along nerve endings to ganglia. Viral infection of neurons leads to extensive intracellular replication, intracellular metabolic derangements, and stress responses(Zhang et al. 2024). Through studies, it is established that HSV infection is the reason for the increase in the intracellular concentration of calcium ions, which can activate intracellular nucleases and proteases and alter cell structure and function(Zaa et al. 2023). Viral infection is also the reason for the activation of an immune response that can induce inflammation in the perineuronal environment and also increase the insult to neurons further(Pistollato et al. 2022). Some viral infections have been proven to last for long periods in neural tissue, and they stay in neuronal tissue for a long period. nerve cells over prolonged durations, and upon weakening of the body’s immunity, reactivation of the virus results in herpes zoster. Research has identified that subsequent to reactivation of VZV, the viral genome transcription and translation processes are enhanced, thus causing extensive replication and release of virus particles. The reactivated virus leads to inflammatory neurological responses and lesions that manifest as neurological signs such as facial palsy(Fu & Pan 2025). Virus particles are also laid down in an unimaginable number of cells in the neurons, viral replication, inducing intracellular metabolic pathology and stress pathways. Replication of the viruses within the neurons has been found to have the capacity to increase intracellular concentrations of calcium ions, intracellular protease and nuclease activation, and structural damage along with loss of cellular function. The viral proteins during viral replication also have the ability to interfere with cellular physiology besides triggering apoptosis(Estevez et al. 2024). In addition, viral replication triggers a robust immune response to cause inflammation along with neuronal tissue damage and thus increasing neurotoxicity. After viral infection of the CNS, RIPK3 kinase has also been found to promote neuronal survival by suppressing excitatory neurotransmission via the mechanism whereby RIPK3 promotes the phosphorylation of calmodulin-dependent protein kinase II (CaMKII), resulting in the activation of cAMP-responsive element-binding proteins and the induction of a neuroprotective transcriptional program with the ability to inhibit deadly glutamatergic signaling(Park et al. 2024).

Facial palsy is a motor disorder of the facial expression group muscles, and its pathogenesis is multifactorial and complex, of which viral infection is an important cause(Alqahtani et al. 2024). It has been proven by research that most peripheral facial palsy and facial neuritis are caused by viral infection.VZV can latently infect the cranial nerves and dorsal root ganglion after primary infection, and when the immune function of the body weakens, the virus is reactivated and causes an inflammatory response in the geniculate ganglion of the facial nerve and results in facial paralysis(Palit et al. 2024). The development of facial paralysis after viral infection is connected with the immune status of the patient, and immunocompromised patients are prone to viral facial paralysis. These patients are prone to viral infection and autoimmune response through defective immune mechanism or immunosuppression and hence facial palsy is more likely(Jain et al. 2024; Yoon et al. 2024). Facial palsy secondary to viral infection has an unpredictable prognosis depending on the virus and severity of infection. Severe damage, delayed recovery and frequent attacks is typical in Hunter syndrome (RHS) due to varicella zoster virus. These patients usually present symptoms of taste impairment, hearing hypersensitivity, tear reduction, and severe pain with severe inflammation and maximum immunoreactivity of the facial nerve canal(Kanerva et al. 2020). Unlike viral facial paralysis, the latter is typically followed by minimal damage, maximum rehabilitation, and minimum recurrence. This facial palsy is also generally devoid of associated taste disturbances and hypersensitivities of hearing and can be managed very well with medications(Ananthapadmanabhan et al. 2022). Clinical epidemiologic studies also found that the idiopathic facial palsy indeed has a high spontaneous rate of recovery since about 70–80% of patients will recover spontaneously within 3 to 4 weeks from the illness onset. The majority of patients with idiopathic facial palsy actually have a high spontaneous rate of recovery. But viral facial palsy is followed by relatively low spontaneous recovery, delayed recovery, and sequelae in some patients. It is known that already about 30% of RHS facial palsy patients fail to regain normal facial function, and the proportion is even greater in diabetic mellitus patients and facial nerve electrogram amplitude < 10%. Age, extent of damage to the axon, oropharyngeal trauma, and multisegmental cranial neuropathies also participate in RHS prognosis. Prognosis of viral facial paralysis will be improved with early detection and treatment(Ghezta et al. 2022).

**Table 1:** Mechanisms of virus-induced facial paralysis

Virus type	Transmission route	Latency site	Infection mechanism	Neural impact
HSV-1	Sensory nerve endings to ganglia	Facial nerve ganglia	Retrograde spread along nerve fibers, inducing inflammation and damage	Inflammation, edema, and compression
VZV	Respiratory droplets	Sensory ganglia	Reactivation and spread along nerve fibers, inducing inflammation and damage	Inflammation, edema, compression, ear and facial pain, vesicles, hearing impairment
SARS-CoV-2	Respiratory droplets and aerosol transmission	primarily infects respiratory andpulmonary tissues	Systemic inflammation affecting nerve blood supply, direct nerve infection	Neuroinflammation, injury, ischemic damage
HIV	Multiple routes (e.g., mother-to-child, blood)	Resting CD4 + T cells, centralnervous system	Immune dysfunction, inducing neuroinflammation	Opportunistic infections and neurological disorders
EBV	Saliva transmission	B cells	Immune-mediated mechanisms inducing facial nerve inflammation and damage	Facial nerve inflammation and injury

### The critical role of immune response in the pathogenesis of facial palsy

Viral infection is one of the leading causes of facial paralysis, and acute inflammatory response to viral infection is a leading pathogen in facial paralysis pathogenesis. When the facial nerve is infected by viruses, facial nerve inflammation and edema can occur due to direct infection of nerve cells or local immune activation(Sawarbandhe et al. 2024). VZV and HSV are prototype causative viruses, which are neuroleptic, have the ability to spread along nerve fibers, and persist in ganglia(Ben Mohamed et al. 2023). Upon viral infection, the intrinsic immunity of the host is triggered to release varied inflammatory mediators. The inflammatory mediators are augmented proinflammatory mediators and augment the acute inflammatory response through activation of downstream signal transduction cascades as well as sensitizing activation and mobilization of inflammatory cells. Release of such inflammatory mediators not only causes inflammation and swelling of the facial nerve but also can cause dysfunction of nerve conduction and finally lead to the development of facial palsy(Bhusal et al. 2021; Chang et al. 2022). NF-κB signal pathway and interferon system are two most important regulation pathways of viral infection-related facial palsy, which are involved in inflammatory response and antiviral immunity profoundly.The activation of NF-κB signal pathway is one of the early mechanisms after viral infection, which might initiate a series of inflammatory mediators. One of the initial responses following viral infection is activation of NF-κB signal pathways, leading to the production of other inflammatory cytokines. Activation of the NF-κB signal pathways also initiates production of vascular endothelial cell adhesion molecules, promoting the migration of inflammatory cells(Liu et al. 2015). The interferon system is a critical part of antiviral immunity to block virus replication through an increase in cellular immunity against the virus. Precocious expression of interferon, critical to preventing viral infection and reduction of facial palsy symptoms, induces cells to synthesize a family of antiviral proteins that inhibit viral translation and replication. Precocious expression of interferon has been demonstrated to reduce viral load and facial nerve edema and inflammation and enhance facial palsy symptoms(Zhang et al. 2025a). Natural immunity is critical to precocious facial palsy. Following viral infection, macrophages and neutrophils rapidly move to the site of infection to discharge mediators of inflammation and produce inflammation and swelling of the facial nerve. Macrophages exacerbate the local inflammatory response by phagocytosing viral particles and cell debris and releasing abundant amounts of inflammatory mediators(Bilge et al. 2022). Neutrophils infect and lyse viral and infected cells with the release of inflammatory mediators by releasing neutrophil elastase and myeloperoxidase, among others(Priya et al. 2024). Naturally occurring immune cells such as dendritic cells and natural killer cells are recruited, identifying viral antigens and initiating an adaptive immune response. Viral nucleic acids are sensed by dendritic cells through the assistance of pattern recognition receptors, which activate the NF-κB pathway and the secretion of inflammatory cytokines. Dendritic cells are further involved in initiating adaptive immune responses through viral antigen presentation and activation of T and B cells(Naranjo-Covo et al. 2025). Natural killer cells, on the other hand, enhance the antiviral activity of the body through the recognition of stress-inducing ligands on infected virus cells and the direct killing of infected cells. capacity. Cooperative function of such natural immune cells not only facilitates regulation of viral infection, but can also influence the course of facial palsy(Wu et al. 2025; Zhu et al. 2025).

T cell and B cell activation is central to the initiation of the adaptive immune response, and T cells also regulate the amount and type of the immune response by secreting many different cytokines. At the onset of facial palsy, T cell-mediated immunity exacerbates the inflammatory process of the facial nerve by the release of proinflammatory cytokines and further damage to the nerve(Luo et al. 2024b). On the other hand, at later stages of facial palsy, prolonged activation and activity of T cells help in the destruction of infected viruses and recovery(Shimizu et al. 2024). B cells destroy viruses mainly by the release of type-specific antibodies against viruses, and the viruses are not permitted to enter further into facial nerve cells(Luo et al. 2024b). B cells can be stimulated and differentiated into plasma cells during facial palsy. At the initial stage of facial palsy, the B cells are activated and differentiate into plasma cells that are held accountable for producing and secreting immunoglobulins in large quantities, and the antibodies have the ability to bind specifically with viral surface antigens to form immune complexes, which are disposed of from the body by the immune system. Virus infection is the strongest causal agent initiating the autoimmune process, and pathogenesis of facial palsy exhibits a very complicated interaction between the virus infection and autoimmune response. When facial nerve cells become infected by a virus, the body’s immune system, in eliminating the virus, may be confused in identifying the facial nerve tissue as a foreign antigen and mount an autoimmune attack(Diggikar et al. 2024). This not only elevates the inflammatory assault within the facial nerve, but also results in permanent damage to the nerves. After viral infection, certain autoantigens of facial nerve cells may have common epitopes with viral antigens, and the immune system can also accidentally injure its own facial nerve cells in the process of attacking viral antigens and cause demyelination of nerve fibers and axonal damage. Memory immune cells can be quickly re-activated when the patient is re-infected with the same causative organism, dividing and differentiating into effector cells rapidly to increase the magnitude and velocity of the immune response. Studies have shown that the presence of immune memory cells can lead to recurrence of facial paralysis, especially in immunocompromised or re-infection(Alqahtani et al. 2024). With re-infection of the body with the same virus, the memory T cells can rapidly differentiate into effector T cells, producing a high level of cytokines to intensify damage to the facial nerve, whereas the memory B cells can rapidly differentiate into plasma cells, producing a high level of antibodies, which can lead to the formation of immune complexes which can lead to the formation and deposition of immune complexes, again damaging the facial nerve tissue(Kathari et al. 2024; Mezni et al. 2024) (Table 2). Therefore, it is of great significance to conduct a comprehensive research on the mechanism and regulation of immune memory to avoid facial paralysis recurrence and improve the prognosis of patients.

Individuals who have immunosuppressed or weakened immune systems are likely to suffer from viral infections as well as autoimmune reactions, significantly increasing the risk of facial paralysis. In idiopathic facial nerve palsy, the immune cells may be inappropriately activated to attack the myelin sheath of the facial nerve and produce demyelination and loss of function, identical to the mechanism in diseases such as Guillain-Barre syndrome(Lu et al. 2025). Additionally, innate immune cells such as neutrophils, macrophages, and NK cells are major players in the cytokine storm. Neutrophils can induce cytokine production by releasing neutrophil extracellular traps, and macrophages are activated to secrete excessive cytokines, inducing excessive tissue damage(Niehues et al. 2024). The research in question has found that, in some facial palsy patients, peripheral blood neutrophil counts and activity are significantly increased and positively correlated with disease severity(Gane et al. 2024; Goto et al. 2024). Cytokine storm is the state in which the body During infection or immune response, the body secretes a huge amount of cytokines excessively, resulting in systemic inflammatory response syndrome and multiple organ dysfunction syndrome. In facial palsy, the overproduction of cytokines activates macrophages and microglia to cause the cells to release more inflammatory mediators. The vicious cycle enhances the inflammatory response(Huang et al. 2025). Cytokine storms also disrupt the integrity of the blood-brain barrier so that peripheral immune cells and inflammatory mediators can flow into the central nervous system to cause neuroinflammation. Overwhelming inflammatory response can lead to direct damage to neurons, inducing apoptosis and dysfunction(de Lima et al. 2025). Research has suggested cytokine storm has been characterized in a variety of illnesses, such as idiopathic multicentric Castleman’s disease, CAR-T cell therapy-induced cytokine storm and sepsis. In these situations, the overproduction of cytokines not only leads to local inflammation, but also results in a systemic inflammatory response, which can even lead to death(Raza et al. 2024; Li et al. 2025b). Dysregulation of the immune system is among the most critical factors involved in facial paralysis pathogenesis. Immunological abnormalities in function may lead to the formation of an autoimmune attack against facial nerve tissue, leading to nerve demyelination and loss of function(Sandhya et al. 2023). Activation of autoreactive B and T cells leads to the production of autoantibodies that attack the nerve tissue and lead to damage of the nerve tissue(Leichtle et al. 2022). Viruses directly infect the facial nerve upon immune activation that leads to immunocompetent cells attacking the nerve tissue(Gaitan-Quintero et al. 2021).

Viruses employ multiple mechanisms to avoid immune surveillance and induce chronic or recurrent facial palsy. Research has shown that HSV-1 evades cGAS-STING-mediated natural antiviral immune function through many proteins. The HSV-1 VP24 protein significantly inhibits IRF3 activation and, in doing so, reduces interferon production(Zhang et al. 2025b). Such an immune evasion mechanism allows the virus to be sustained in the host, resulting in chronic or recurrent facial palsy. Direct evidence for expression of immune evasion mechanisms in facial palsy etiology is given by viral repression of host antiviral capabilities and induction of viral replication(Chweya et al. 2019). Cytomegalovirus escaping immunorecognition by inducing a range of proteins suppressing the host type I interferon function has been reported from correlational studies. These mechanisms, in addition to maintaining the virus in the host, are also responsible for inducing repetitive damage to the facial nerve that leads to chronic or recurrent facial paralysis(Kathari et al. 2024).

**Table 2:** Key immunological events in the pathogenesis of facial palsy

Stage	Immune cells	Main function	Outcomes and impacts
Early stage of facial palsy	T cells	Secretion of pro-inflammatory cytokines	Exacerbation of facial nerveinflammation and worseningof nerve damage
Early stage of facial palsy	B cells	Production of specific antibodies	Neutralization of viruses and prevention of further invasion
Late stage of facial Palsy	T cells	Clearance of viral infection	Promotion of disease recovery
Late stage of facial palsy	B cells	Secretion of immunoglobulins and complexes	Clearance of residual viruses
Autoimmune reaction	T cells/B cells	Misidentification and attack on facial nerve tissues	Demyelination and axonal damage of nerve fibers
Immune memory cells	Memory T cells/Memory B cells	Rapid activation upon re-infection	Aggravation of nerve damage and increased risk of recurrence

## Synergistic mechanisms of viral infection and immune response

Viral replication in the facial nerve elicits a host immune response. Viral replication by-products and signaling molecules released from damaged cells are able to guide immune cells like macrophages, T cells, and B cells to the infected site to migrate in. Activation and recruitment of these immune cells constitute the initial phase of the immune response for virus clearance and repair of injured tissues(Kim et al. 2016; Bruurs et al. 2023). Although the initial function of the immune response is to remove the virus, an overactivated immune response has been shown to lead to neurological damage. It has been proven that cytokines and free radicals released from immune cells after viral infection have been found to directly damage nerve cells. These cytokines are able to trigger neuroinflammation resulting in edema and facial nerve compression to impact nerve function(Mangus et al. 2015). Additionally, overactivation of the immune cells is able to trigger an autoimmune response, wherein the immune system attacks the facial nerve by mistake, leading to demyelination of nerve fibers and axonal degeneration. Having the virus within the facial nerve will keep activating the immune system, with a consequence being long-term inflammation and ongoing nerve destruction. It has been shown by research that in Hunter’s syndrome induced by varicella zoster virus, intense inflammation and strong immune response within facial neural tube lead to delayed and incomplete facial paralysis recovery(Chung et al. 2023).

Viruses modulate the activity of immune cells by a variety of different mechanisms, and hence affect the duration and intensity of the immune response. Laboratory tests have proven that neurotropic viruses such as VZV and HSV can infect the facial nerve and produce local inflammation accompanied by activation of immune response. Viruses are able to inhibit the activation of B cells and T cells, which interferes with the immune system(Kong et al. 2024; Li et al. 2025a). HSV-1 viruses were discovered in other research to evade immune detection by inhibiting the type I interferon signaling pathway. Viruses induce apoptosis of immune cells and suppress immune cell counts(Jellinge et al. 2021; Zakharzhevskaya et al. 2025). VZV viruses were discovered in research to induce apoptosis of microglia, thereby weakening the immune system. Viruses control cytokines produced by immune cells and thus control the inflammatory process. An effective immune response can kill the virus and minimize the virus-induced neural damage; however, an excessive or inadequate immune response can lead to viral latency and relapse. Immune systems clear viruses by using varied mechanisms, such as cellular immunity and humoral immunity, where T cells recognize and destroy virus-infected cells infected with the virus, and B cells produce a virus that can be recognized and treated by the immune system. T-cells recognize and destroy virus-infected cells, and B-cells produce neutralizing antibodies to inhibit virus entry into host cells(Sivanandham et al. 2020; Boeren et al. 2023). Administration of high doses of glucocorticosteroids in the initial stages has been shown by research to reduce inflammation, prevent the toxicity of viruses and ganglia and nerve fiber loss, and reduce neural edema to minimize tissue damage(Chomont et al. 2018; Heldman et al. 2022).Immunodeficiency leads to viral latency, and research has shown that immunocompromised patients are at risk of viral relapse, which initiates facioscapular degeneration and virus relapse.Virus relapse, which in turn causes neurologic presentation like facial palsy. Also, viruses have the ability to enter the latent stage by evading immune monitoring and reactivate when the immunity is compromised(Moffat et al. 2007; Rolle & Olweus 2009). There is a delicate balance between the immune system’s capacity to defend and damage the facial nerve following a viral infection. A proper immune response eliminates the virus and defends the nerve tissue, while an overactive immune response can cause nerve damage. Safety mechanisms include antiviral immunity that destroys the virus through cellular and humoral immune processes and restricts direct viral damage to nerve tissue(Pavlou et al. 2024); and anti-inflammatory responses, where the immune system protects nerve tissue by reducing inflammatory responses through release of anti-inflammatory cytokines. However, mechanisms of injury cannot be overlooked either. Cytokine storms can cause hyperimmune responses, which lead to extreme neuroinflammation and tissue damage; autoimmune responses can cause the immune system to mistakenly identify neural tissue as a foreign antigen, which triggers an autoimmune response that can lead to neural demyelination and dysfunction(Vazquez et al. 2024); further, in immune-mediated neurologic injury, immune cells such as neutrophils and macrophages can release inflammatory mediators that can directly damage neural tissue Neutrophils can directly damage neural tissue by releasing enormous inflammatory mediators. Neutrophils also damage nerve cell and tissue through release of excess inflammatory mediators such as reactive oxygen species and proteases, while macrophages damage nerves by releasing inflammatory mediators and directly phagocytosing nerve tissues(Vazquez et al. 2024).

**Fig. 1:**
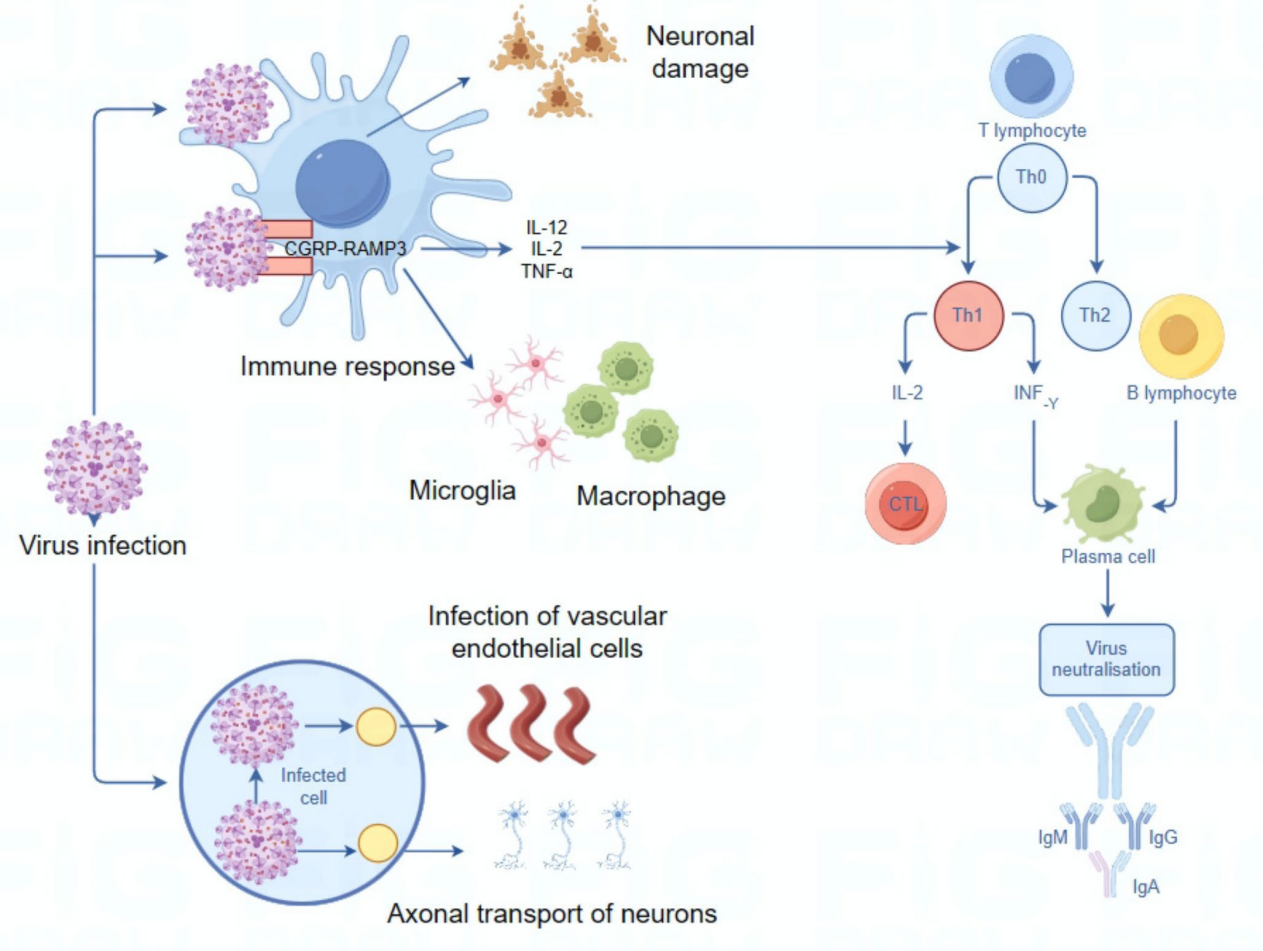
Key nodes and mechanisms of the viral-immune-neural axis (By Figdraw)

The interaction amongst viral infection, immune response, and nerve trauma is highly complex. Immune response may be triggered by viral infections, subsequently affecting the nervous system’s form and function. Viral infections may invade the CNS through varied pathways, i.e., either by direct invasion of vascular endothelial cells or through neuronal axonal fibers. Once the virus has penetrated the CNS, microglia in the brain parenchyma, macrophages and dendritic cells in the choroid plexus, meninges, and perivascular space initiate an immune response to clear the invading virus. But hyperimmune state may trigger neuroinflammation and neuronal injury in a chronic manner(Pavlou et al. 2024). Several molecules and pathways are implicated in viral infection in the virus-immunity-neuroaxis interaction. CGRP-RAMP3 neuroimmunity axis is essential for mice antiviral immunity. It was found that viral clearance process mediated by Th1 cells involves expression of CGRP, and RAMP3 gene-deficient mice had impaired Th1 cell differentiation and reduced viral clearance(Yin et al. 2025) (Fig. 1). In addition, autophagy receptor SHISA9 regulates microglia and astrocyte immune response during CNS infections, and inhibits inflammatory response by interacting with non-anchored ubiquitin chains IKKi-bound to support IKKi degradation(Hall et al. 2025).

## Conclusions and future perspectives

In this study, we investigated the pathogenesis of facial palsy, elucidated the pivotal role of viral infection in the pathogenesis of facial palsy and the role of immune response as a pivotal mechanism on facial palsy, and revealed the synergistic effect between viral and immune response simultaneously. These findings provide new ideas for clinical diagnosis and treatment of facial palsy, suggesting that we need to combine viral testing with immune examination in clinical diagnosis to develop more specific treatment measures. However, many research gaps, such as heterogeneity of immune responses induced by various viruses and other special mechanisms, are still to be explored further. Subsequent studies should take advantage of full multidisciplinary cross-over, for instance, synergy between genomics, proteomics and immunology, to evaluate new biomarkers and targets of therapy. While this is done, research and development and clinical evidence of innovative therapeutic approaches, such as biological agents, monoclonal antibodies and cell therapy, should be accelerated to pursue actively the opportunity of personalized care and precision medicine in facial palsy. Finally, there can be no disregard of viral infection and immunologic response’ cooperative functions in the disease mechanism of facial palsy. We call for sustained basic research and clinical effort in a bid to improve the prevention, diagnosis, and treatment of facial palsy and patients’ advantage accordingly.

## Electronic supplementary material

Below is the link to the electronic supplementary material.

Supplementary Material 1

## Data Availability

No datasets were generated or analysed during the current study.
