# Supplementary material for: The synergistic role of viral infection and immune response in the pathogenesis of facial palsy
Source: J Neurovirol. 2025 May 15;31(3):208–18. doi: 10.1007/s13365-025-01258-7 (PMC12356718; doi:10.1007/s13365-025-01258-7)
Supplement: Supplementary file 1 — Supplementary Material 1 [file 13365_2025_1258_MOESM1_ESM.docx]

Supplementary material

**Author contributions**  AW provided funding, conceptualized the work, wrote the original draft. WX designed figures, reviewed, edited. JZ reviewed, provided critical feedback, and prepared the final manuscript.

**Data availability** No datasets were generated or analysed during the current study.

**Competing interests** The authors declare no competing interests.
